# Supplementary material for: Intermittent hypoxia mediated by TSP1 dependent on STAT3 induces cardiac fibroblast activation and cardiac fibrosis
Source: eLife. 2020 Jan 14;9:e49923. doi: 10.7554/eLife.49923 (PMC6992386; doi:10.7554/eLife.49923)
Supplement: Supplementary file 1. [file elife-49923-supp1.docx]

**RT-PCR primers**

| Gene | Stand | Sequence |
| --- | --- | --- |
| Mouse Acta2 | forward | CATCTTTCATTGGGATGGAGTCAG |
|  | reverse | CCCCTGACAGGACGTTGTT |
| Mouse Col1a1 | forward | GCTCCTCTTAGGGGCCACT |
|  | reverse | CCACGTCTCACCATTGGGG |
| Mouse Postn | forward | TGGTCACTTCACGCTCTTTG |
|  | reverse | TGGAGATACTGTCCCCTTCG |
| Mouse Thbs1 | forward | GCAAAGACGTCGATGAGTGC |
|  | reverse | CGGTTTGCACACCTGTTTGT |
| Mouse Tnfa | forward | TAGCCAGGAGGGAGAACAGA |
|  | reverse | TTTTCTGGAGGGAGATGTGG |
| Mouse Stat3 | forward | GCAATACCATTGACCTGCCG |
|  | reverse | TCAAACGTGAGCGACTCAAAC |
| Mouse 18S | forward | GGAAGGGCACCACCAGGAGT |
|  | reverse | TGCAGCCCCGGACATCTAAG |
